# Working Memory Capacity Limits Memory for Bindings

**DOI:** 10.5334/joc.86

**Published:** 2019-09-19

**Authors:** Klaus Oberauer

**Affiliations:** 1 University of Zurich, Department of Psychology – Cognitive Psychology, Zürich, CH

**Keywords:** Working memory, Mathematical modelling, Short-term memory

## Abstract

I propose that the capacity of working memory places a specific limit on the maintenance of temporary bindings. Two experiments support this *binding hypothesis*: Participants remembered word lists of varying length. When tested on a randomly selected word, their error rates increased with the length of the list, reflecting a limited capacity for short-term maintenance. This increase in errors was predominantly due to binding errors: People confused the correct word with other words of the current memory list, but very rarely with words not in the list. The frequencies of response choices were analyzed through two measurement models – one based on the assumption of discrete memory states, one on the assumption of continuous memory strength – that capture memory for items and for bindings in separate parameters. Increasing memory set size impaired binding memory but not item memory, supporting the binding hypothesis.

People have no difficulty understanding a short sentence of four to six words and repeating it verbatim after a second, but when the sentence becomes long and convoluted, they begin to struggle. This limit on our ability to remember and process complex information reflects the capacity limit of working memory. Differences between people in working-memory capacity is strongly correlated with their ability to understand language, to reason, and to learn, and with their general intelligence (Conway, Kane, & Engle, 2003; Daneman & Merikle, 1996; Gathercole, Pickering, Knight, & Stegman, 2004).

The capacity limit of working memory is usually described as a limit on how much information can be maintained over a few seconds. All information? No: Here I show that the capacity limit of working memory (WM) pertains to the short-term maintenance of bindings but not items. Memory for items is the ability to remember which individual items (e.g., words, visual objects) have occurred in the relevant episode (e.g., in the memory set the person is asked to hold in mind). Memory for bindings is the ability to remember relations between items (e.g., which object has been presented together with which word), or relations between items and their context (e.g., which object has been presented in which location, or which word has been presented in which serial position of a list).

My conjecture is that WM capacity reflects a specific limit on our ability to establish and maintain temporary bindings (e.g., a new order of words, or a new spatial arrangement of known objects). Forming and upholding such bindings is essential for building new structural representations, which underlie reasoning and language comprehension. Hence, the capacity of WM limits the complexity of new relational representations, and this explains why measures of WM capacity are excellent predictors of individual differences in fluid intelligence (Oberauer, 2017; Oberauer, Süß, Wilhelm, & Sander, 2007).

So far, this *binding hypothesis* of WM capacity has received support from findings about individual differences in cognitive abilities (Oberauer, 2005; Wilhelm, Hildebrandt, & Oberauer, 2013): Indicators of a person’s ability to maintain temporary bindings are highly correlated with scores on standard WM tests, and with fluid intelligence. Here I provide an experimental test of the binding hypothesis. The primary experimental finding demonstrating the limited capacity of WM is the set-size effect: As the number of items to be held in WM increases, performance decreases (Bunting, Cowan, & Saults, 2006; Grenfell-Essam & Ward, 2012; Ma, Husain, & Bays, 2014; Oberauer & Kliegl, 2001). A prediction from the binding hypothesis is that set size affects primarily memory for bindings, not items.

The present experiments investigate the effect of set size on immediate memory for items and for bindings. Participants tried to remember lists of words presented across a row of frames. Participants were tested on a randomly chosen list position, and asked to selected the word in that position from a set of response candidates arranged randomly on the screen. The response set contained the correct word, other words from the current list, and new words. Item memory – defined in the context of these experiments as memory for which items have been in the current list – is sufficient to discriminate between correct and other list words on the one hand, and new words on the other. Binding memory – defined as memory for which word was in which list position – is needed to discriminate the correct word from other list words. I predict that increasing memory set size (i.e., the number of list words) affects predominantly binding memory, and not – or much less – item memory.

The difficulty of a memory test also depends on the number of responses the person must choose from, the response set size (RSS). The binding hypothesis implies that, despite their limited WM capacity, people have good memory for which words have been in the current list even for large memory sets. This should enable them to limit their effective response set to those response candidates that come from the current memory set. Hence, the binding hypothesis entails the prediction that performance is affected by the number of words in the response set that come from the current list (RSS_List_) but much less, if at all, by the number of new words in the response set (RSS_New_). The *n*-alternative forced-choice (*n*-AFC) test procedure used in the present experiments enables me to control RSS_List_ and RSS_New_ largely independently of the size of the memory set.

## Method

### Experimental Design

I tested these predictions with two experiments varying memory set size (2, 4, 6, or 8 words) and test condition. Test condition was defined by the composition of the response set, coded [RSS_List_, RSS_New_]: [1,1], [2,0], [2,2], [4,0], [4,4], [6,0], [6,4], [8,0], [8,4]. As a tenth test condition I included a recall test in which participants had to type the probed word. Because memory set size constrains RSS_List_, crossing these two variables left some design cells structurally empty: With memory set size 2, only the first 3 RSS conditions could be realized; with memory set size 4, the first five RSS conditions were possible; with memory set size 6, the first 7 RSS conditions were possible, and only memory set size 8 afforded all nine RSS conditions. Hence, there were 3 + 5 + 7 + 9 = 24 *n*-AFC conditions plus 4 recall conditions.

I ran the experiment in two versions, one using a large pool of words as materials, so that each word was used only rarely in a trial, and one with a small pool of 16 words that were re-used frequently. The two versions place different demands on item memory: In the *large-pool experiment*, item memory required discriminating the words seen in the present trial from new words never seen in the entire experiment. This could be accomplished by an episodic-memory record of the words experienced in the experimental setting, without distinguishing between the current trial and previous trials. In the *small-pool experiment*, item memory required discriminating the words in the current list from the words seen in other recent trials. Moreover, with the large pool, recall differs from *n*-AFC because it requires the additional ability to recover the identity of a word from a potentially distorted memory trace (a process sometimes called “redintegration”; Hulme et al., 1997; Lewandowsky, 1999; Schweickert, 1993). With the small pool, that demand becomes trivial by the frequent repetition of the same small set of words. Once a person has learned the 16 words in the pool, recall effectively becomes a 16-AFC test. Therefore, I expect that in the small-pool experiment – but not in the large-pool experiment – recall performance will be predictable from *n*-AFC performance on the assumption that in a recall test, people use the memory set as the response set.

### Participants

Each experiment enrolled 20 students of the University of Zurich for three one-hour sessions. They were reimbursed by partial course credit or 45 Swiss Francs (~ 45 USD). I chose the sample size because it is sufficient to detect medium to large effects in within-subjects designs, and because memory set-size effects are known to be large. The use of Bayesian statistics means that the sample size could have been increased in case of ambiguous evidence (Rouder, 2014), but this was not necessary.

### Materials

The large pool consisted of 1198 German nouns with a length of less than 16 characters, drawn at random from the data base Semantischer Atlas (Schwibbe, n.d.). The small pool for each participant was a new random set of 16 words drawn from the large pool. For the large-pool experiment, the words for each memory list were drawn at random without replacement from the pool. The response set consisted of a subset of these memory words and a set of new words, also drawn without replacement from the pool. When the pool was exhausted, it was re-instated in full, and sampling resumed as before. In this way, words could be used a second time only after all words from the pool have been used once, so that a new word in the set of response options, if it has occurred before in the experiment at all, had occurred many trials ago. For the small-pool experiment, the list words and the new words to be included in the response set were sampled from the pool without replacement in each trial; after each trial the pool was re-instated in full, so that the same words could be (and usually were) re-used on the next trial.

The list items included in the response set were chosen by first ordering all list items by their positional distance to the tested item, from smallest to largest. From that ranked list I chose the first RSS_List_ items. This procedure avoids a confound between memory set size and the average positional distance of response options to the tested item. If response options were drawn at random from the list without constraint, their average distance from the tested item would increase with set size. Because people tend to confuse list items more with closer than with more distant neighbors on the list (Hitch, 1974; Lee & Estes, 1977), this confound would lead to an underestimation of the set-size effect.

### Procedure

**Figure 1:**
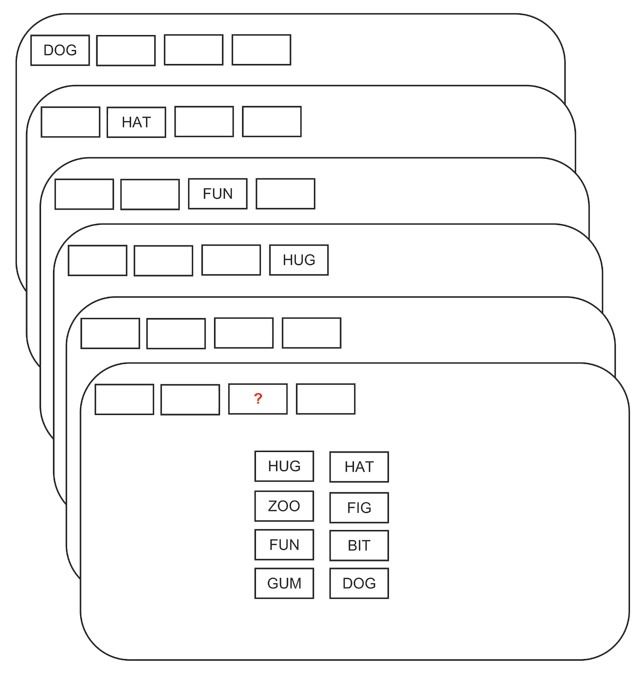
Flow of events in a trial with memory set size 4 and RSS = [4,4].

Figure 1 shows the procedure of a typical trial. Each trial commenced with the presentation of 2 to 8 rectangular frames, corresponding to the trial’s list length, in a row from left to right in the upper quarter of the screen. After 0.5 s the first word was presented in the left-most frame for 0.9 s, followed by 0.1 s during which the frame turned blank again, before the next word was presented in the next frame to the right. A 1.0 s study-test delay during which all frames were empty followed the last list word. Then a question mark appeared in one of the frames, indicating the serial position (and spatial location) of the tested word. For *n*-AFC test trials, the response options were displayed at the same time in a random arrangement of two columns and as many rows as needed (depending on the RSS) centered in the lower two-thirds of the screen. Participants were instructed to select the correct word by clicking on it with the mouse. For recall trials, the query “Please type the tested word” was displayed instead, and participants typed their response, confirming it by pressing the Return key. After a 2.0 s inter-trial interval during which the screen was blank, the next trial started.

The partial crossing of memory set size (4 levels) with test condition (9 compositions of *n*-AFC response sets plus the recall test) resulted in 28 design cells (24 *n*-AFC and 4 recall tests). All 28 conditions were presented in random order. In the first session, participants did 28 practice trials (one from each condition), followed by 7 blocks of test trials. Each block consisted of 28 trials, one per condition. In each of the two subsequent sessions, they did 5 warm-up trials (drawn at random from the 28 conditions) followed by 8 test blocks.

### Data Analysis

#### Descriptive Analysis

The descriptive analysis used a Bayesian hierarchical logistic regression predicting the number of correct responses in the *n*-AFC tests by memory set size, RSS_List_, and RSS_New_. In addition to the fixed effects of these predictors the model included a random effect of subject (i.e., random intercept) as well as terms for individual differences in the sizes of all main effects and two-way interactions (i.e., random slopes). I implemented the model with the R package *brms* (Bürkner, 2017). Evidence for the effect of each predictor was assessed by comparing the full model to a model excluding the fixed main effect of that predictor. I used the Bayes factor for model comparison, calculated through the bridge sampler (Gronau, Singmann, & Wagenmakers, 2018) included in the *brms* package.

The Bayes factor depends on the priors of the effect sizes in the models, in particular the effect size that is included in one model and removed in the other: A more uninformative prior favors more strongly the null hypothesis over the alternative hypothesis. As there is not yet an established default prior for logistic models, I developed a default prior on standardized effect sizes based on the rationale for choosing default priors for linear models developed by Rouder, Morey, Speckman, and Province (2012) together with a sensitivity analysis exploring the effect of a range of plausible priors on the Bayes factors; see Appendix A for details.

#### Measurement Models

**Figure 2:**
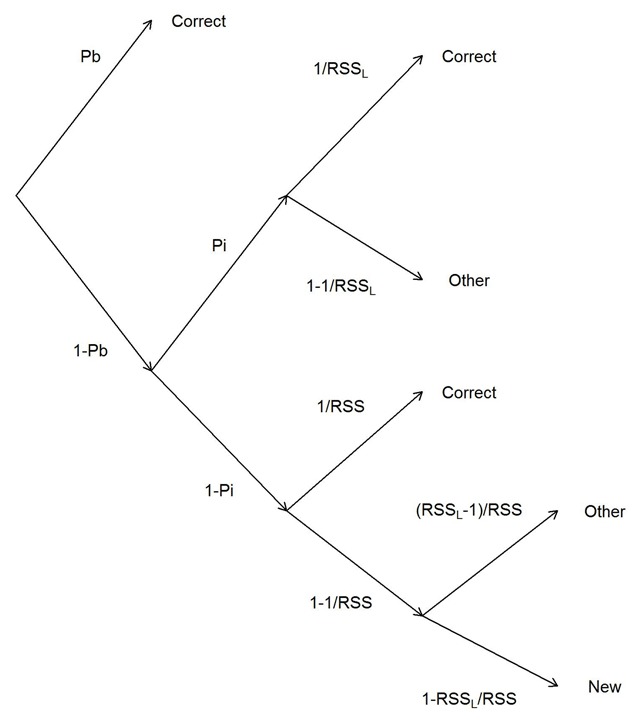
Structure of the multinomial process tree (MPT) model; at the end of each branch is the predicted response category (correct, other list word, or new word). Pb is the probability of remembering the target’s item-position binding; Pi is the probability of remembering which items have been in the current memory list. RSS_L_ refers to the size of the subset of the response set that consists of current list items; RSS refers to the total size of the response set.

To obtain separate measures of item memory and binding memory I used two measurement models, a multinomial process-tree (MPT) model building on the assumption of discrete memory states (Riefer & Batchelder, 1988), and a memory measurement model (MMM) building on the assumption of continuously varying memory strength (Oberauer & Lewandowsky, in press). The process tree of the MPT model is depicted in Figure 2. For an *n*-AFC test of memory for a given list position, the word bound to that position is available with probability *Pb* (i.e., the probability of an intact word-position binding at the tested location). If that binding is available, the correct response is always given. With probability 1-*Pb*, the binding is not available, and in that case the person has item memory about which words were in the list with probability *Pi*. If item memory (but no binding memory) is available, they choose a response from the response candidates that come from the current list, guessing among them with equal probability. When item memory is unavailable, they choose with equal probability among all response candidates.

The MMM is based on the assumption that all response candidates receive different degrees of activation reflecting the strength of evidence from memory in favor of choosing them (Oberauer & Lewandowsky, in press). All candidates receive baseline activation *B*. All list words receive additional activation *A*, the parameter reflecting the strength of item memory. The correct word, by virtue of being bound to the probed position, receives additional activation *C*, where *C* reflects the strength of binding memory. The predicted activation values of the three response categories are:

\begin{array}{l}
{A_{correct}} = B + A + C\\
{A_{other}} = B + A\\
{A_{new}} = B
\end{array}

The probability of a response in category *j* is given by Luce’s choice rule:

P(j) = \frac{{{n_j}{A_j}}}{{\sum\nolimits_j {{n_j}{A_j}} }},

with *n_j_* = 1 for correct responses, *n_j_* = RSS_List_-1 for other list words, and *n_j_* = RSS_New_ for new words.

To make the MMM identifiable, one of the three parameters (*A, B*, or *C*) has to be fixed to provide the scale of the other two. When introducing the MMM we fixed *B* to 0.1 (Oberauer & Lewandowsky, in press). Here I found it useful to estimate *B* freely to capture the difference between the two experiments: The “new” words were much less new in the context of the small-pool experiment than in the context of the large-pool experiment, and that should be reflected in a larger estimate of *B* in the former. Therefore, here I fixed the mean of *C* across conditions to 10 (an arbitrarily chosen value). Hence, any effect of the experimental conditions on *C* is expressed as the deviation of the *C* parameter in each condition from 10.

Both measurement models predicted the frequencies of responses in the three categories for each of the 24 *n*-AFC conditions. Each model had one parameter reflecting item strength and one reflecting binding strength; these parameters were modelled as linearly dependent on memory set size.

The models were implemented as Bayesian hierarchical models in Jags (Plummer, 2016). The models estimated group-level estimates for the mean and the regression slope (i.e., the effect of mean-centered memory set size) on each of the two memory parameters. The group-level estimates were the means of normal distributions describing the distribution of individual subject’s parameter values. After confirming that the models provide a reasonable description of the data (see Appendix B), I used the posterior distributions of the group-level slope estimates to ask whether set size had an effect on the item-memory parameter, on the binding-memory parameter, or on both.

## Results

**Figure 3:**
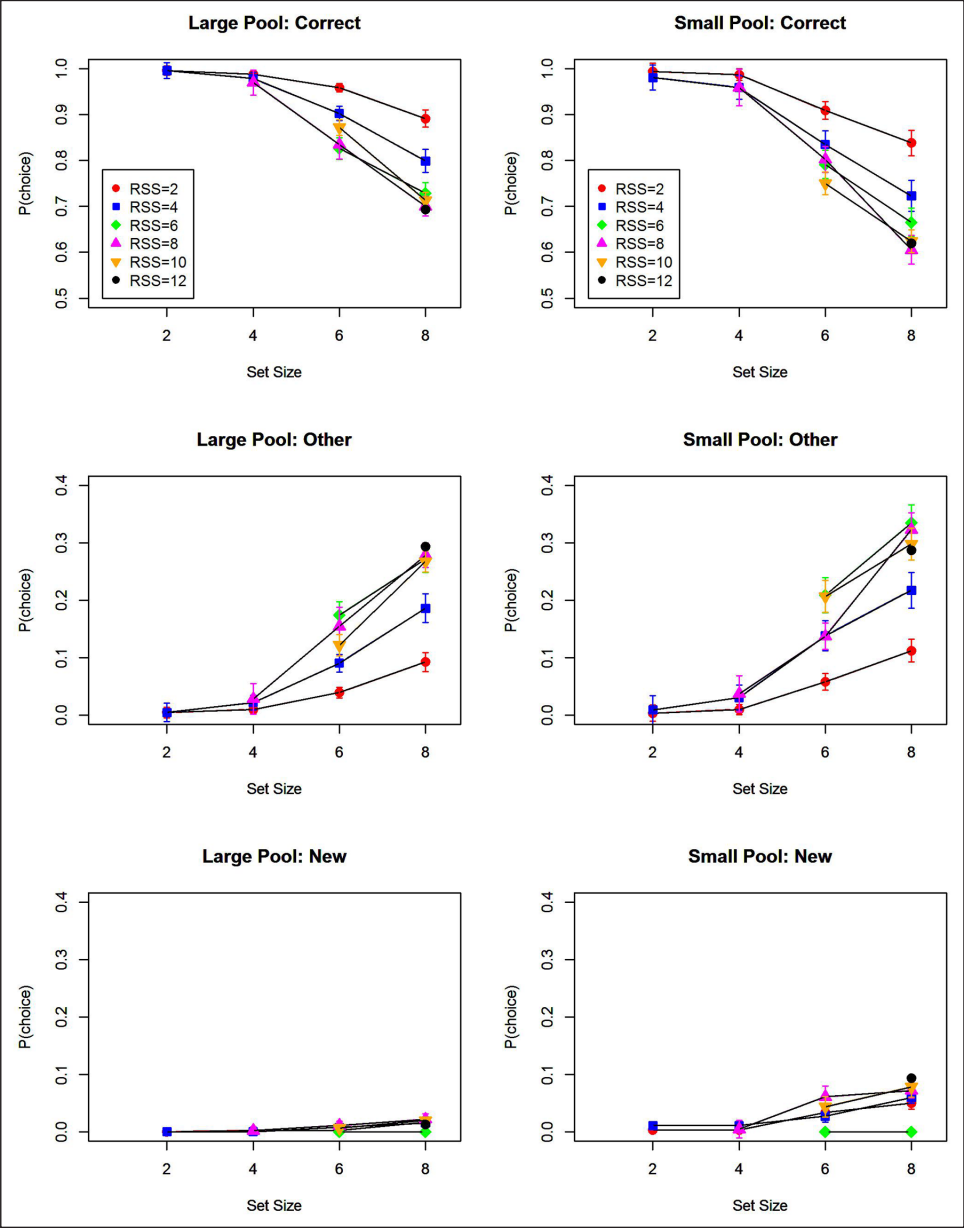
Proportion of correct responses, of responses selecting another than the correct list word, and of responses selecting a new word. Separate lines represent different response set sizes. Error bars are 95% confidence intervals corrected for within-subjects comparisons (Bakeman & McArthur, 1996).

Figure 3 shows the proportions of the three response categories – correct, other list words, and new words – as a function of memory set size and total response set size. The set-size effect on accuracy was nearly exclusively due to an increase of selecting other list items. Participants in the large-pool experiments hardly ever selected new words; they did so somewhat more often in the small-pool experiment, but still at a much lower rate than they selected other list words. Hence, the increase of error rates with increasing memory set size was predominantly an increase in binding errors, not item errors. This is the error distribution expected from the binding hypothesis.

**Figure 4:**
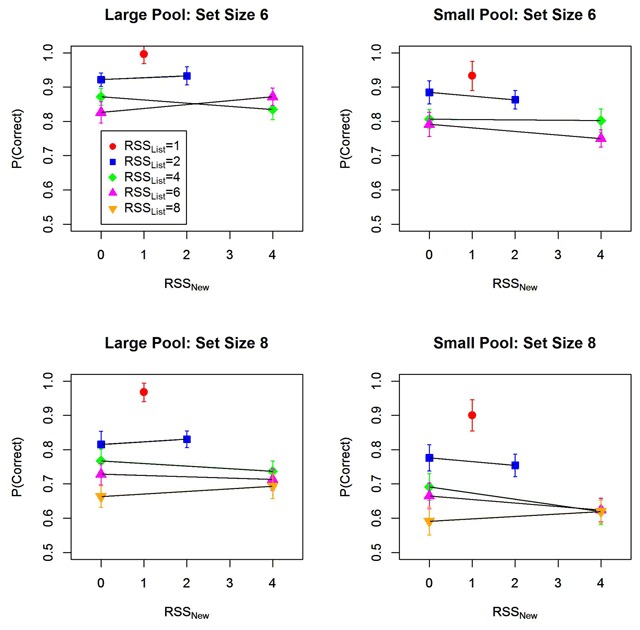
Proportion of correct responses as a function of memory set size (6 vs. 8), as well as the size of the response subset consisting of list words (RSS_List_) and the response subset consisting of new words (RSS_New_). Error bars are 95% confidence intervals corrected for within-subjects comparisons (Bakeman & McArthur, 1996).

Figure 4 decomposes the effect of response set size into the effects of RSS_List_ and of RSS_New_. Only RSS_List_ had a strong and consistent effect on performance, confirming the assumption that participants are very good at limiting their selections to words from the current list. Table 1 summarizes the Bayes factors for the main effects of set size, RSS_List_, and RSS_New_.

**Table 1:** Bayes Factors for Logistic Models.

	Memory Set Size	Response Set Size List	Response Set Size New

Large-Pool Experiment	1.07 × 10^10^ [1.00–1.77 × 10^10^]	1.85 × 10^11^ [1.16–3.36 × 10^11^]	0.37 [0.048–0.49]
Small-Pool Experiment	4.14 × 10^10^ [3.29–7.21 × 10^10^]	2.65 × 10^8^ [0.78–2.69 × 10^8^]	14.2 [1.8–16.4]

*Note*: The Bayes factor reflects the strength of evidence for keeping the effect in question in the model over excluding it. It expresses the factor by which we should multiply the ratio of our prior probabilities assigned to the competing models to obtain our ratio of posterior probabilities. Bayes Factors are based on Cauchy priors on standardized effect sizes with a scale of .353; the range of Bayes Factors for scales between 0.25 and 3.0 obtained from the sensitivity analysis is given in brackets.

**Figure 5:**
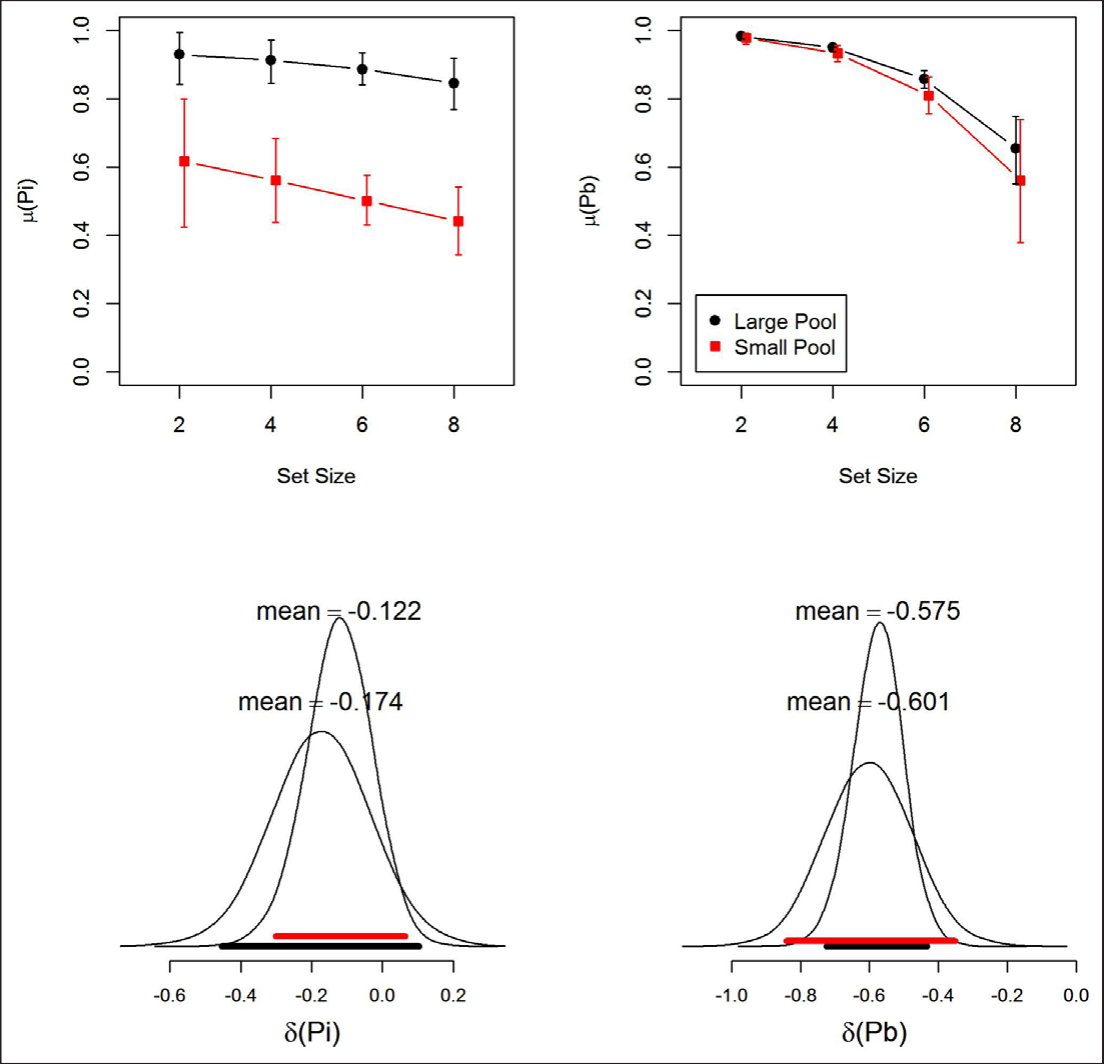
Top panels: Group-level parameter estimates (means of the posterior distribution) for item memory (Pi) and binding memory (Pb) from the MPT model. Black markers represent the large-pool experiment; red markers the small-pool experiment. Error bars are 95% highest-density intervals of the posteriors (Kruschke, 2011). Bottom panels: Posterior distribution of the slope of the linear effect of (mean-centered) memory set size on group means of Pi and Pb. Broad horizontal bars depict the 95% highest-density intervals (black for the large-pool experiment; red for the small-pool experiment).

Figure 5 shows the parameter estimates from the MPT model. The binding-memory parameter *Pb* declined with set size in both experiments; accordingly, the posterior distribution of the set-size slope on *Pb* was unambiguously in the negative range. The item-memory parameter *Pi* also appears to decline over set sizes. However, the posterior slope on *Pi* spans both sides of zero, with a substantial proportion in the positive range (10.5% and 9.0% in the large-pool and small-pool experiment, respectively). Whereas binding memory was comparable in both experiments, item memory was better in the large-pool experiment, showing that participants found it easier to discriminate between list words and words they had never seen in the experiment than to discriminate between list words and other words used frequently on previous lists.

**Figure 6:**
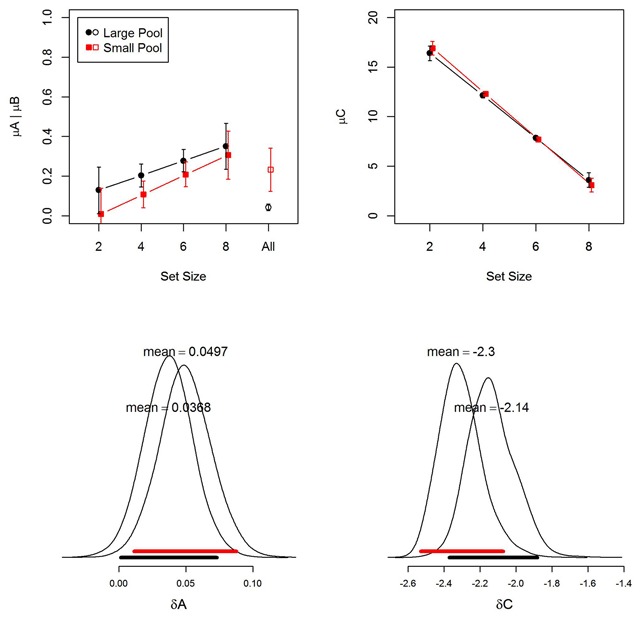
Top panels: Group-level parameter estimates (means of the posterior distribution) for item memory (A) and binding memory (C) from the memory measurement model (MMM). The upper-left panel also shows the B parameter, which was the same for all set sizes (“All” on the x-axis). Black markers represent the large-pool experiment; red markers the small-pool experiment. Error bars are 95% highest-density intervals of the posteriors (Kruschke, 2011). Bottom panels: Posterior distribution of the slope of the linear effect of (mean-centered) memory set size on group means of A and C. Broad horizontal bars depict the 95% highest-density intervals (black for the large-pool experiment; red for the small-pool experiment).

Figure 6 shows the parameter estimates from the MMM. The binding-memory parameter *C* declined with set size in both experiments, as reflected in the negative set-size slopes on *C*. In contrast, the item-memory parameter *A* slightly increased with set size. The two experiments differed primarily in the size of the baseline activation *B*. Unsurprisingly, *B* was higher in the small-pool experiments, in which the “new” words were repeated many times throughout the experiment.

On the assumption of discrete memory states, the capacity of WM is often expressed as an estimate of the number of items remembered (Adam, Vogel, & Awh, 2017; Cowan, 2001; Zhang & Luck, 2008). For *n*-AFC tests of a single item, this estimate is obtained from

K = N\frac{{P(correct) - g}}{{1 - g}},

with *K* for the number of remembered items; *N* for the memory set size, *P(correct)* for proportion of correct responses, and *g* for the chance of guessing the correct response. The chance of guessing depends on our assumptions about the capacity limit. Therefore, we can ask which assumptions result in more consistent estimates of *K*.

**Figure 7:**
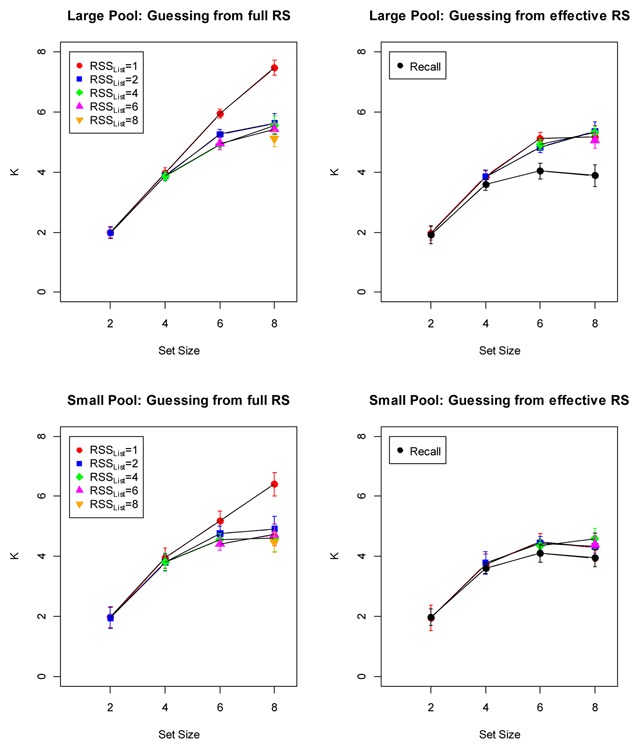
Estimates of capacity (K) on the assumption of discrete-state memory. The left panels show K estimates for a capacity limit on item memory; the right panels show K estimates for a capacity limit on binding memory. All panels show estimates from n-AFC tests as a function of the size of the response subset consisting of list words (RSS*_List_*); the right panels additionally show estimates from the recall test. Error bars are 95% confidence intervals corrected for within-subjects comparisons (Bakeman & McArthur, 1996).

If the capacity limit is a limit on all information remembered about an item, then we distinguish a state of remembering the tested item (leading to a correct response) and a state of no information about the item, in which case the person guesses with equal probability from the entire response set: *g* = 1/RSS. By contrast, if we assume that WM capacity places a limit on binding memory but not item memory, then we distinguish a state of remembering the item-position binding (leading to a correct response) and a state of not remembering the binding, but still having item memory available to restrict the effective response set to the candidates from the current list. On this assumption, guessing chooses each candidate from the current list with equal chance, and *g* = 1/RSS_List_. Figure 7 shows the *K* values calculated in both ways. The *K* estimates based on complete loss of information, resulting in uninformed guessing, diverge for different levels of RSS_List_, whereas those based on loss of only binding information converge better. Hence, if we want to describe the capacity limit of WM in terms of a discrete number, then describing it as a maximum number of item-context bindings leads to more consistent values than describing it as a maximum number of items. In theories that assume a discrete capacity limit on the number of items that can be held in WM (Adam et al., 2017; Cowan, 2001), that limit should be constant across variations of RSS. Therefore, theorists endorsing this assumption should characterize capacity as limiting the number of items for which bindings to their context can be maintained, rather than a limit on remembering the item per se.

When assuming that WM capacity limits binding but not item memory, then we can estimate *K* also for the recall test: Based on item memory, the person can construct an effective response set consisting only of the items in the current memory set. Assuming for simplicity that item memory is perfect, we can calculate *K*, using *g* = 1/*N*. This estimate is included in the right-hand panels of Figure 7. For the large-pool experiment this estimate was lower than that from the *n*-AFC tests, whereas for the small-pool experiment the estimates from the two test procedures converged. This could reflect the fact that with a large pool, the recall test involves the additional demand of recovering the word’s identity from a partially degraded representation retrieved from memory. In the small-pool experiment, where all the words from the pool are arguably well learned, that demand becomes trivial, so that recall becomes effectively an *n*-AFC test with all pool items as nominal memory set, and the current list items as effective memory set.

## Discussion

The present experiments provide evidence for the binding hypothesis: WM capacity is a limit on the maintenance of bindings, not items. This evidence is most clearly expressed in the parameters of the two measurement models: Memory set size had a strong negative effect on memory for binding. In contrast, the effect on memory for items was weak at best, and inconsistent between the two measurement models. Hence, whether one thinks of WM in terms of discrete states of remembering or not, or in terms of continuous memory strength, measures of binding memory showed a pronounced set-size effect, as expected from a capacity limit, whereas memory for items did not. This is not to say that memory for items was perfect – in the small-pool experiment it clearly was not. Yet, whatever limits item memory does not do so more strongly with larger set size, and hence cannot be described as a capacity limit.

These observations about model parameters reflect a distinct pattern in the data: Errors increased with memory set size, and that increase was nearly entirely due to binding errors (i.e., confusing a list item with another list item). Moreover, memory performance declined with an increase in RSS_List_, but was little, if at all, affected by RSS_New_. I next discuss some questions and objections that I expect readers to entertain.

### Does Item Memory Reflect Long-Term Memory?

The fact that item memory did not decline with memory set size could be explained by assuming that episodic long-term memory (eLTM) provides sufficient information about which items were in the current list to prevent most item errors. This is a possibility, and it does not contradict the binding hypothesis. The binding hypothesis states that item memory is unaffected by the capacity limit of WM – this could be because WM itself has a large, perhaps unlimited capacity to remember recent items. Alternatively, this could be because eLTM provides sufficient item memory, but not binding memory, to meet the demands of WM tests. The latter assumption is plausible for the large-pool experiment, because here item memory merely requires remembering which words have been seen in the context of the experiment – an ability usually attributed to eLTM. It has been shown that memory for trial-unique items is much better than expected from common estimates of WM capacity (Endress & Potter, 2014), and this memory feat could be attributed to information in eLTM about which items have been seen in the experiment. It is also known that one form of item memory – familiarity – far outlasts individual trials of short-term memory tests (Monsell, 1978). Familiarity would be sufficient to discriminate old from new words in the large-pool experiment. In the small-pool experiment, item memory requires discriminating current-list words from words used repeatedly in preceding lists. This could be accomplished by eLTM through associations of words to trial contexts. Alternatively, it could be accomplished by WM through temporary bindings of words to the current trial context, or by maintaining the words from the current trials active, while de-activating all words at the end of a trial.

Whichever mechanism is responsible for maintaining information about individual items, it is powerful enough to keep item memory at a high level, undiminished for set sizes up to 8 items, larger than any estimate of WM capacity ever published. This is so under test conditions that are typical for tests of WM (i.e., a presentation rate of one item per second followed by an immediate test; a small pool of repeatedly used items). It is conceivable that, if item memory is provided by eLTM, there is still a capacity limit for items in WM, but then this capacity limit does not become manifest in conditions of typical WM tasks. As such, the assumption of such a capacity limit on item memory does not contribute to explaining people’s performance limitations in typical WM tasks. For reasons of parsimony we should not make such an assumption.

### Why Did Many Previous Tests of Working Memory Show Limited Item Memory?

The memory set-size effect on performance has been demonstrated with every task used for studying WM (Oberauer et al., 2018), and these tasks typically don’t show a set-size effect only on binding errors. For instance, in serial recall, both order errors and item errors (i.e., extra-list intrusions and, if permitted, omissions) increase with set size (e.g., Grenfell-Essam & Ward, 2012). In change-detection tests of visual WM, performance declines with set size regardless of whether the changes affect the identity of an item in the array, so that it could be detected on item memory alone, or affects only the relations between items and spatial locations, so that memory for item-locations is needed (e.g., Donkin, Tran, & Le Pelley, 2015; Rouder et al., 2008). In the continuous-reproduction task of visual WM, in which features of array items are reproduced on a continuous response scale, a substantial proportion of errors does not reflect erroneous retrieval of the wrong array item (i.e., a binding error), but rather appear unrelated to any item in the current array, and the prevalence of these errors increases strongly with memory set size (e.g., Adam et al., 2017). Why is that so?

I propose that the apparent capacity limit on item memory arises from related but slightly different causes in different test forms. Consider serial recall, the most often used test of verbal and spatial WM. The WM system uses each list position to retrieve the item bound to it. As set size increases, the bindings of items to list positions is impaired, so that it becomes harder to discriminate the target item in a given position from other list items. Thereby, the retrieved representation of the target item is increasingly distorted by being blended with representations of other list items (Oberauer, Farrell, Jarrold, Pasiecznik, & Greaves, 2012). This leads to confusion of the target item with other list items (i.e., order errors), but also to confusions with similar extra-list items in the experimental vocabulary (i.e., extra-list intrusions), as well as failures to recover any legitimate retrieval candidate (i.e., omission errors). In the present *n*-AFC tests, omission errors are impossible, and extra-list intrusions are rare because the new words included in the response set are rarely very similar to a list item. Therefore, impaired bindings translate nearly exclusively into confusions with other list items.

Consider next tests of visual WM, in which arrays of simple visual stimuli varying on one or two feature dimensions (e.g., colors, orientations) are to be remembered. Because of the low dimensionality of the stimulus set, there is only a small set of stimuli that are easily discriminable (e.g., the eight cardinal orientations, and an about equal number of color categories, see Bae, Olkkonen, Allred, & Flombaum, 2015). Therefore, even when the items in a given array are well discriminable from each other, they are poorly discriminable from most stimuli that were not in the array but are very similar to one of the array items. This poses a challenge for item memory – whether based on WM or eLTM – that becomes more severe with larger set sizes. For instance, if a person can discriminate eight color categories and is asked to remember an array of eight well-discriminable colors, there will be hardly any color category left that was not in the current array. Hence, distinguishing colors in the current array from colors not in the array becomes extremely difficult. As a consequence, item memory is expected to be poor. Therefore, when binding memory fails – as it will at larger set sizes – then errors are not likely to look like binding errors in the context of good item memory (i.e., confusions with other items in the current array) but rather like errors arising from poor binding memory and poor item memory (i.e., apparently random guesses).

## Conclusions

The capacity limit of WM is a limit on bindings, not items. Demonstrating this requires measuring item and binding memory separately. This can be accomplished by measurement models using separate parameters for item and binding memory. In addition, we need a well-defined (ideally, experimenter-controlled) response set consisting of highly discriminable stimuli, so that response candidates from the current memory set are well discriminable from new candidates.

## Data Accessibility Statement

All raw data and R code are available at the OSF: osf.io/qy5sd.

## Additional Files

The additional files for this article can be found as follows:

10.5334/joc.86.s1 Appendix A. Priors for Logistic Regression.

10.5334/joc.86.s2 Appendix B. Observed Response Proportions and Posterior Predictives of the Measurement Models.
