# Supplementary material for: Working Memory Capacity Limits Memory for Bindings
Source: J Cogn. 2019 Sep 19;2(1):40. doi: 10.5334/joc.86 (PMC6753309; doi:10.5334/joc.86)
Supplement: Appendix A. — Priors for Logistic Regression. [file joc-2-1-86-s1.pdf]

Appendix A: Priors for Logistic Regression

Priors should reflect what we know about the effect in question before looking at the data. When specific knowledge about the studied effect is not available, priors should incorporate more general knowledge about the sizes of similar effects. Rouder et al. (2012) have proposed a default prior for the General Linear Model based on general knowledge about the distribution of standardized effect sizes in the social sciences. This default prior is a Cauchy prior on the standardized effect size (i.e., the standardized deviation of each condition mean from the grand mean), scaled to values between ½ and 1. No such default priors have yet been proposed for logistic models. Gelman, Jakulin, Pittau, and Su (2008) propose a Cauchy with scale = 2.5 for logistic regression, but this proposal is intended for parameter estimation, not model comparison, and is therefore designed to be fairly uninformative (i.e., reflect a broader prior distribution than warranted by our knowledge). I explored the distribution of predicted probabilities that a logistic model with an intercept of zero and a single effect of a binary predictor (coded as -0.5 and 0.5) generates when its effect size is Cauchy distributed with a scale between 0.25 and 3. Scales > 0.75 predict an excess of extreme probabilities (i.e., values close to 0 or 1), in stark departure from what is known about typical effect sizes. I therefore chose a Cauchy prior with scale = 0.35 as the default prior, which generates a realistic distribution of probabilities. I also explored the sensitivity of the Bayes factors to the scale in the range from 0.25 to 3.

References

Rouder, J. N., Morey, R. D., Speckman, P. L., & Province, J. M. (2012). Default Bayes factors for ANOVA designs. *Journal of Mathematical Psychology, 56*, 356-374.

Gelman, A., Jakulin, A., Pittau, M. G., & Su, Y.-S. (2008). A weakly informative default prior distribution for logistic and other regression models. *The Annals of Applied Statistics, 2*, 1360-1383. doi:10.1214/08-AOAS191
