# Supplementary material for: Working Memory Capacity Limits Memory for Bindings
Source: J Cogn. 2019 Sep 19;2(1):40. doi: 10.5334/joc.86 (PMC6753309; doi:10.5334/joc.86)
Supplement: Appendix B. — Observed Response Proportions and Posterior Predictives of the Measurement Models. [file joc-2-1-86-s2.pdf]

Appendix B: Observed Response Proportions and Posterior Predictives of the Measurement Models

Figures B1 to B3 present the proportions of correct responses, responses choosing other list words, and responses choosing new words in the *n*-AFC tests for each design cell in the large-pool experiment. The figures also show the mean posterior predictives from the two measurement models for these data. Figures B4 to B6 show the data from the small-pool experiment together with the posterior predictives of the measurement models.

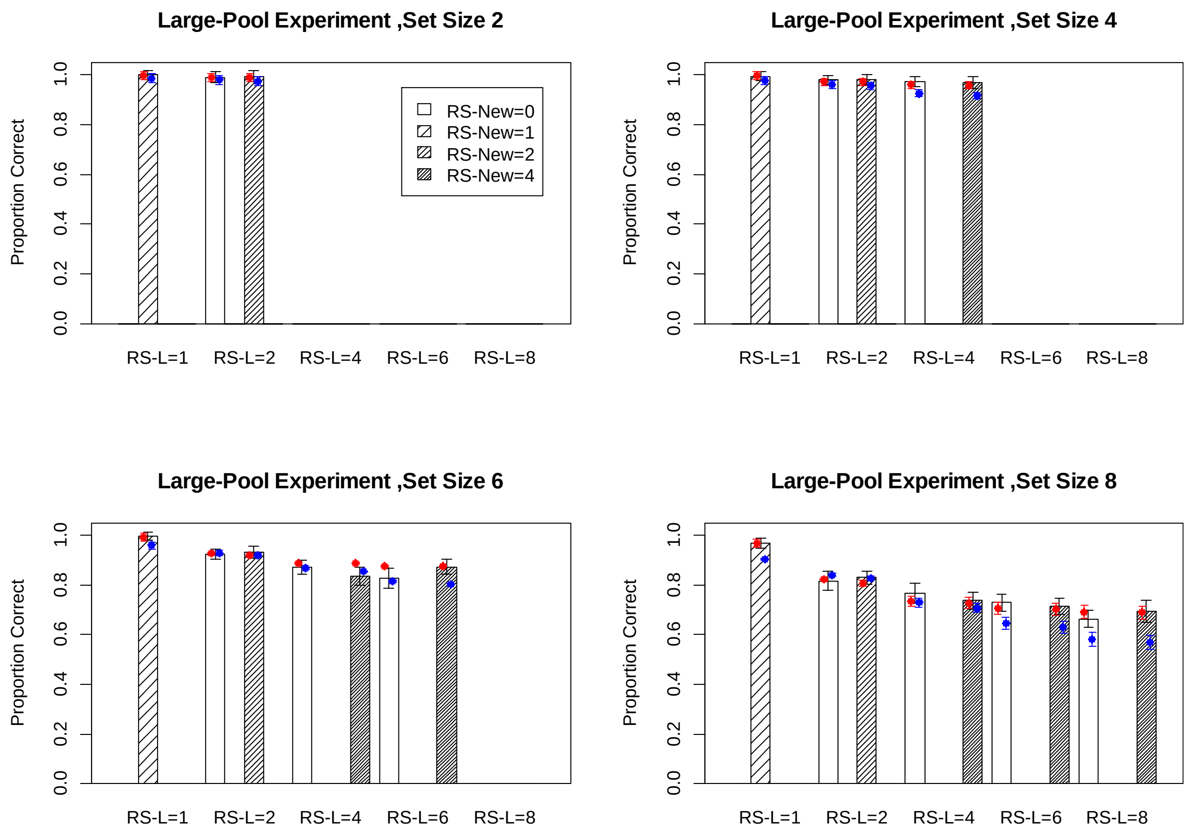

Fig. B1. Proportion of correct responses in the large-pool experiment. Each panel shows the observed proportions of responses as bars; error bars are 95% confidence intervals for within-subjects comparisons (Bakeman & McArthur, 1996). The predictives of the MPT model (in red) and of the MMM (in blue) are the means over subjects of the mean posterior predictive distributions (depicted as dots), with the 95% confidence intervals computed from the standard deviation of the means of individual subjects in the same way as for the observed data.

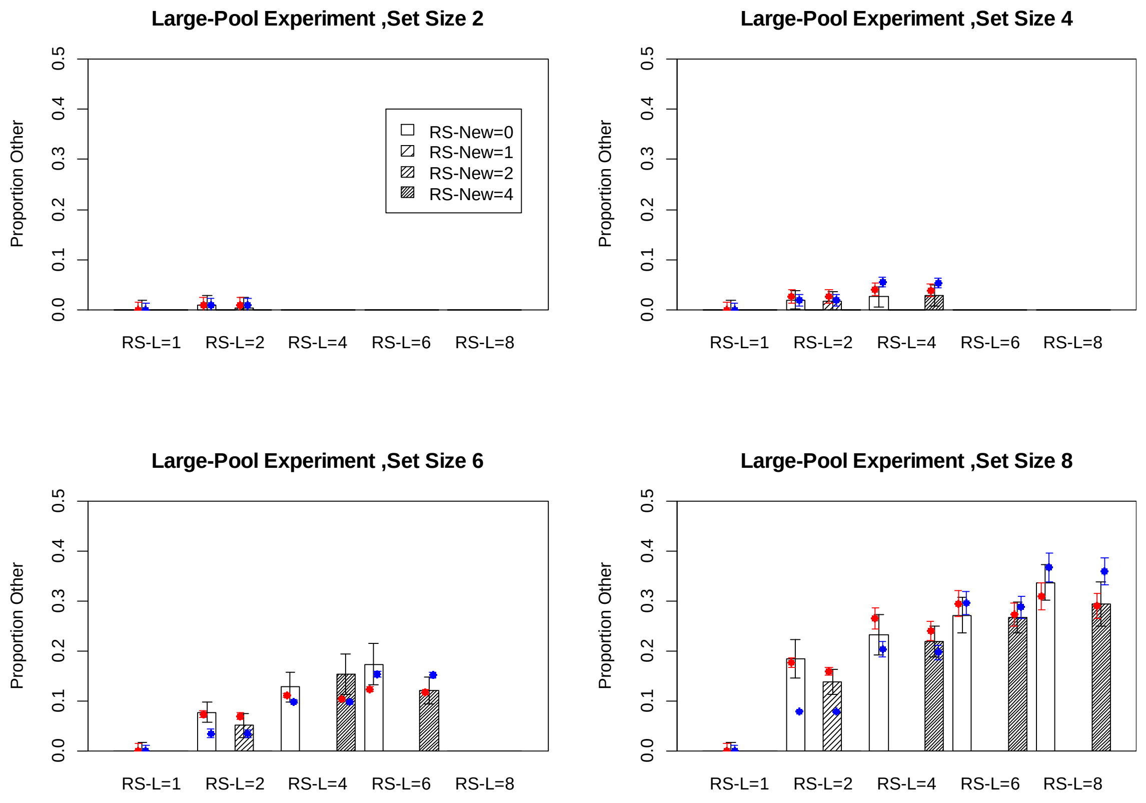

Fig. B2. Proportion of other list item responses in the large-pool experiment. Each panel shows the observed proportions of responses as bars; error bars are 95% confidence intervals for within-subjects comparisons (Bakeman & McArthur, 1996). The predictives of the MPT model (in red) and of the MMM (in blue) are the means over subjects of the mean posterior predictive distributions (depicted as dots), with the 95% confidence intervals computed from the standard deviation of the means of individual subjects in the same way as for the observed data.

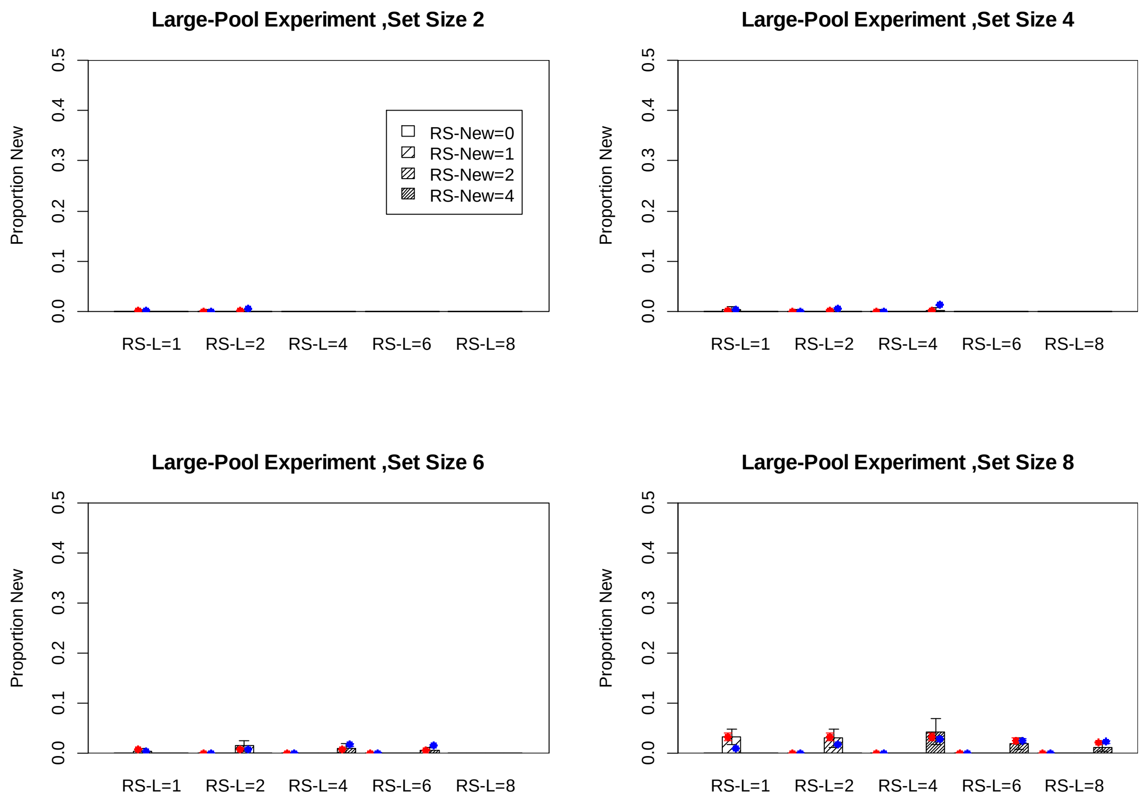

Fig. B3. Proportion of new responses in the large-pool experiment. Each panel shows the observed proportions of responses as bars; error bars are 95% confidence intervals for within-subjects comparisons (Bakeman & McArthur, 1996). The predictives of the MPT model (in red) and of the MMM (in blue) are the means over subjects of the mean posterior predictive distributions (depicted as dots), with the 95% confidence intervals computed from the standard deviation of the means of individual subjects in the same way as for the observed data.

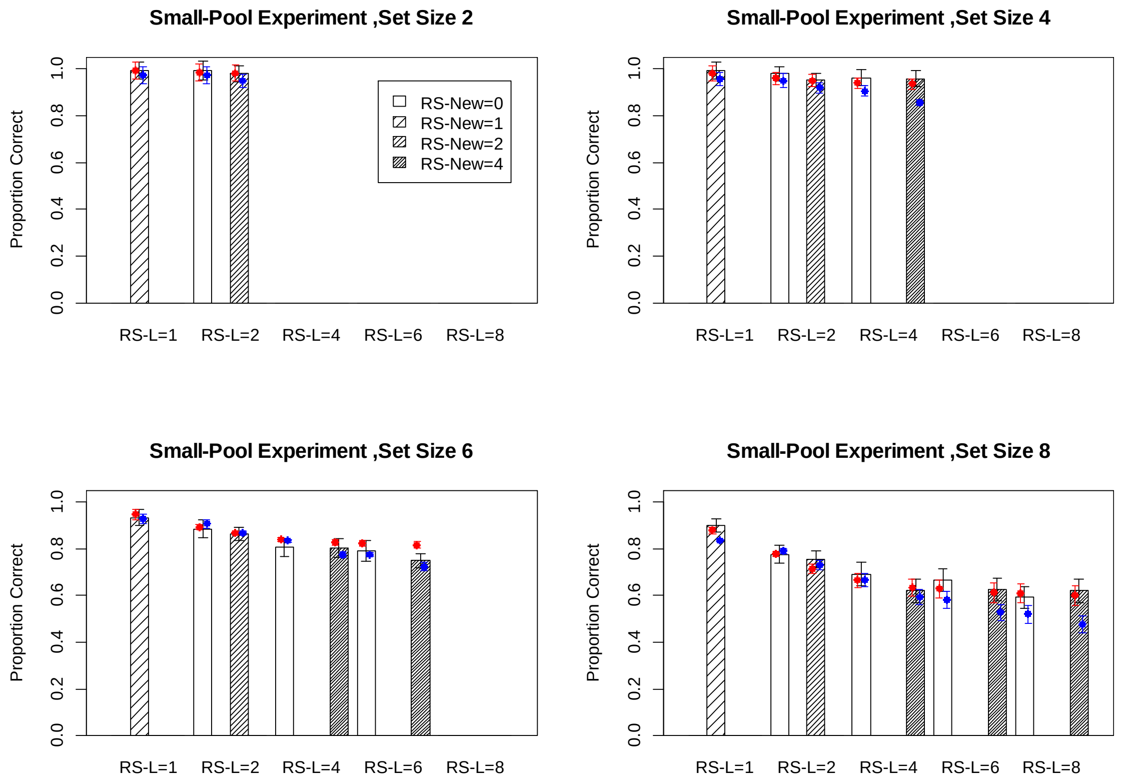

Fig. B4. Proportion of correct responses in the small-pool experiment. Each panel shows the observed proportions of responses as bars; error bars are 95% confidence intervals for within-subjects comparisons (Bakeman & McArthur, 1996). The predictives of the MPT model (in red) and of the MMM (in blue) are the means over subjects of the mean posterior predictive distributions (depicted as dots), with the 95% confidence intervals computed from the standard deviation of the means of individual subjects in the same way as for the observed data.

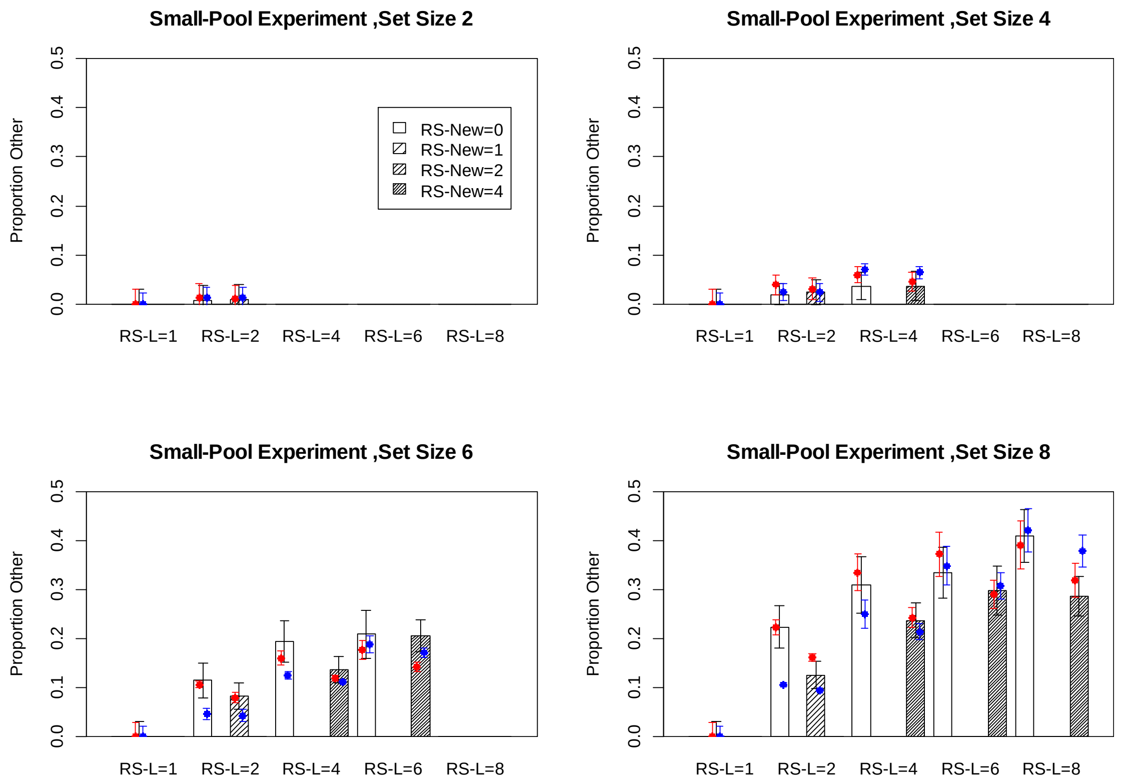

Fig. B5. Proportion of other list item responses in the small-pool experiment. Each panel shows the observed proportions of responses as bars; error bars are 95% confidence intervals for within-subjects comparisons (Bakeman & McArthur, 1996). The predictives of the MPT model (in red) and of the MMM (in blue) are the means over subjects of the mean posterior predictive distributions (depicted as dots), with the 95% confidence intervals computed from the standard deviation of the means of individual subjects in the same way as for the observed data.

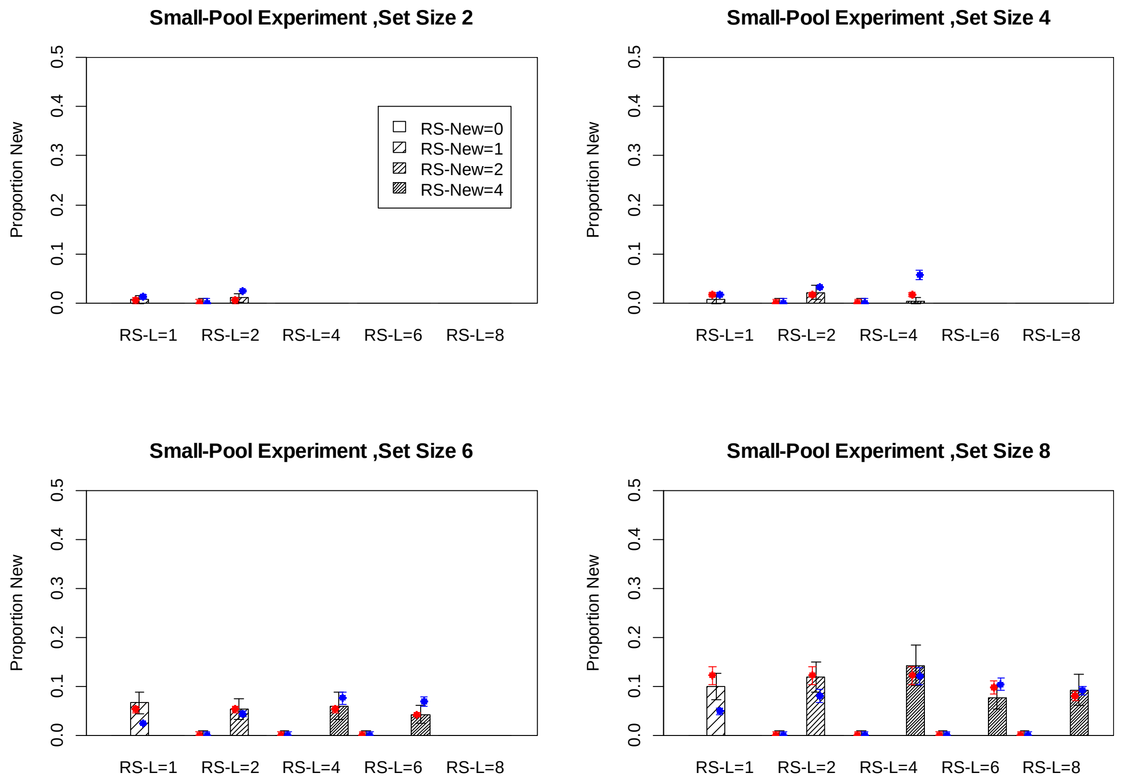

Fig. B6. Proportion of new responses in the small-pool experiment. Each panel shows the observed proportions of responses as bars; error bars are 95% confidence intervals for within-subjects comparisons (Bakeman & McArthur, 1996). The predictives of the MPT model (in red) and of the MMM (in blue) are the means over subjects of the mean posterior predictive distributions (depicted as dots), with the 95% confidence intervals computed from the standard deviation of the means of individual subjects in the same way as for the observed data.
